# Establishment of a novel lupus mesenteric vasculitis mouse model and exploration of its exacerbation mechanisms

**DOI:** 10.3389/fimmu.2026.1886325

**Published:** 2026-09-09

**Authors:** Maki Fujishiro, Kunihiro Hayakawa, Yuko Kataoka, Marina Shinoura, Hiroyuki Tomita, Yuna Saito, Takuya Nishi, Keigo Ikeda, Kenji Takamori, Iwao Sekigawa, Shinji Morimoto

**Affiliations:** 1 Institute for Environment and Gender-Specific Medicine, Graduate School of Medicine, Juntendo University, Chiba, Japan; 2 Department of Internal Medicine and Rheumatology, Juntendo University Urayasu Hospital, Chiba, Japan

**Keywords:** intestinal microbiota, lupus mesenteric vasculitis, propionate, systemic lupus erythematosus, Toll-like receptor 7

## Abstract

Lupus mesenteric vasculitis (LMV) is a gastrointestinal lesion primarily caused by vasculitis associated with systemic lupus erythematosus (SLE). In our preliminary experiments, we found that NZBWF1 (BWF1) mice, a spontaneous SLE mouse model, developed bloody stools when treated with imiquimod, a Toll-like receptor (TLR) 7 agonist. Therefore, in this study, we investigated the effects of the continuous application of a TLR7/8 agonist resiquimod (R848) on the gastrointestinal tract in BWF1 mice. To address this, 8-week-old female BWF1 mice were treated with R848 three times weekly for up to 6 weeks. We found that R848-treated mice developed intestinal vasculitis. In addition, these mice exhibited significantly increased serum autoantibody levels and accelerated SLE pathology. Because these symptoms closely resemble human LMV pathology, we established this as a novel inducible LMV mouse model. To elucidate the mechanisms underlying the onset and exacerbation of LMV, we analyzed changes in the intestinal environment. LMV-induced mice showed altered intestinal microbiota and increased fecal levels of propionic acid, an intestinal microbiota metabolite. To investigate the effect of bacteria on LMV pathogenesis, we treated LMV-induced mice with broad-spectrum antibiotics and found that this treatment tended to suppress LMV pathology early in the induction period. Furthermore, propionate administration appeared to attenuate intestinal vasculitis in these mice. Thus, we found that increased intestinal propionate contributes to attenuating LMV pathology. In conclusion, we established an inducible LMV mouse model by R848 treatment of BWF1 mice. Using this induced LMV mouse model, the pathophysiology of LMV can be analyzed in detail. Additionally, our findings suggest that bacteria influence the induction and exacerbation of LMV pathogenesis, and that intestinal propionate may be involved in maintaining homeostasis. In the future, this LMV mouse model may contribute to the development of new diagnostic and therapeutic methods.

## 1 Introduction

Lupus mesenteric vasculitis (LMV) is a gastrointestinal lesion that is associated with systemic lupus erythematosus (SLE). LMV is rare complication of SLE and frequently presents with acute abdominal pain (1–3). The symptoms of LMV, such as abdominal pain, nausea, vomiting, ascites and diarrhea, are similar to those of infections enteritis, and there are no distinctive clinical findings (4). Therefore, early diagnosis and treatment are difficult, especially when it presents as an initial manifestation of SLE (2). Diagnosis primarily involves blood tests, imaging, and pathology (4). Pathologically, vasculitis is observed in the subserosa of the small intestine (5). However, because pathological evaluation is highly invasive and difficult to perform, the diagnosis is mainly based on a comprehensive assessment of imaging findings, such as intestinal wall thickening, intestinal wall enhancement (target sign), and engorgement of mesenteric vessels (comb sign) (2). Furthermore, while LMV is effectively treated with steroids and immunosuppressants (1), there are concerns about the side effects of their long-term use (6). Therefore, clarifying the mechanisms of LMV onset and exacerbation could lead to the development of LMV-specific diagnostic and therapeutic methods in the future. However, currently, the mechanisms underlying LMV onset and exacerbation remain elusive, as no mouse models that exhibit its pathology have been established.

SLE is a prototypical autoimmune disease characterized by multi-organ damage caused by the production of diverse autoantibodies, deposition of immune complexes in tissues, and infiltration of inflammatory cells (7). SLE is triggered not only by genetic factors but also by environmental factors such as ultraviolet radiation, viral infections, and female hormones. Regarding environmental triggers, antiviral defense mechanisms are closely associated with Toll-like receptors (TLRs). Specifically, TLR7 functions as a sensor for single-stranded RNA (ssRNA) viruses, such as influenza virus and coronavirus, as well as self-derived ssRNA (8). In addition, aberrant TLR7 signaling plays a crucial role in the pathogenesis of SLE in both humans and mice (9–11). Consistent with these findings, topical application of a TLR7 agonist induces SLE-like autoimmune pathology in wild-type mice (11). Based on these findings, we previously demonstrated that continuous treatment of NZBWF1 (BWF1) mice, a spontaneous SLE model, with the TLR7 agonist imiquimod (IMQ) led to the early onset of lupus nephritis (12). Interestingly, during these experiments, we discovered that the IMQ-treated mice developed markedly bloody stools. Therefore, in this study, based on our observation in IMQ-treated mice, we investigated the underlying mechanisms of bloody stools using an R848-induced mouse model. Specifically, we aimed to elucidate whether excessive TLR7/8 signaling induced by continuous application of the TLR7/8 agonist R848 causes intestinal lesions, and whether this gastrointestinal phenotype reflects the pathogenesis of LMV.

## 2 Materials and methods

### 2.1 Mice and *in vivo* treatment

All mouse experiments were approved by and performed in accordance with the Institutional Animal Care and Use Committee of Juntendo University (approval numbers: 310237, 2020094, 2021091, 2022070, 2023082, 2024110, 2025048) and conducted in accordance with the ARRIVE (Animal Research: Reporting of *In Vivo* Experiments) guidelines. Female NZBWF1 (BWF1), male BXSB, and female MRL/MpJ-*Fas^lpr^* (MRL/*lpr*) mice were purchased from Japan SLC (Hamamatsu, Japan). Female BALB/c mice were purchased from Jackson Laboratory Japan (Yokohama, Japan). BXSB mice and MRL/*lpr* mice were utilized as SLE model mice in addition to the BWF1 strain, with BALB/c mice serving as wild-type controls. To normalize the intestinal flora, mice were housed in our animal facility for at least 4 weeks prior to the start of the experiments. All mice were randomly assigned to groups and housed at 3–6 mice per cage. BWF1 mice used in this study were selected based on the absence of increased serum anti-dsDNA antibody titers at 8 weeks of age. The number of mice in all experiments is provided in the figure legends.

To induce autoimmunity via TLR7 overactivation, resiquimod (R848), a synthetic TLR7/8 agonist, was primarily utilized. BWF1 mice were treated with 10 µg of R848 (Enzo Life Sciences, Farmingdale, NY) three times per week via topical application to the skin on one side of the pinna for up to 6 weeks. Similarly, R848 was administered to other strains starting at the following ages: 1) BXSB mice from 6 weeks of age, 2) MRL/*lpr* mice from 5 weeks of age, and 3) BALB/c mice from 8 weeks of age. The treatment duration was 3 weeks for BXSB and MRL/*lpr* mice, and 6 weeks for BALB/c mice. In addition, IMQ cream (Beselna Cream; Mochida Pharmaceutical, Tokyo, Japan), a selective TLR7 agonist, was utilized as an alternative to R848. IMQ cream (1.25 µL) was applied to the skin on one side of the pinna three times per week used for 6 weeks.

To investigate the role of the intestinal microbiota, antibiotics were administered to deplete intestinal bacteria. The antibiotic mixture consisted of vancomycin (0.5 g/L; FUJIFILM Wako, Osaka, Japan), neomycin (1.0 g/L; FUJIFILM Wako), ampicillin (1.0 g/L; Nacalai Tesque, Kyoto, Japan), and metronidazole (0.5 g/L or 1.0 g/L; FUJIFILM Wako) in drinking water (13). The administration of antibiotics was initiated 3 weeks before R848 application. Metronidazole was administered at a concentration of 0.5 g/L during the first week as an acclimatization period, and the concentration was subsequently increased to 1.0 g/L from the second week onwards.

Sodium propionate (150 mM; Sigma-Aldrich Japan, Tokyo, Japan) was dissolved in the drinking water and provided *ad libitum* (14). The sodium propionate solution was administered concurrently with the R848 application for 3 weeks.

### 2.2 Clinical examination

Serum anti-dsDNA antibody levels (specific for IgG) were measured using a mouse anti-dsDNA antibody ELISA kit (FUJIFILM Wako Shibayagi, Shibukawa, Japan). Total IgG levels were determined using an ELISA kit (Thermo Fisher Scientific, Waltham, MA). Fecal occult blood was measured by the *o*-tolidine colorimetric method. Platelet counts were determined using whole blood anticoagulated with EDTA-2Na. Urinalysis was performed to qualitatively assess proteinuria. Proteinuria was measured using urine test strips (Uropaper III, EIKEN, Tokyo, Japan) and scored as − (<15 mg/dL), ± (15–30 mg/dL), 1+ (30–100 mg/dL), 2+ (100–300 mg/dL), 3+ (300–2000 mg/dL), or 4+ (>2000 mg/dL), according to the manufacturer’s scoring system.

Anti-neutrophil cytoplasmic antibodies (ANCA) were evaluated as follows: bone marrow was collected from BALB/c mouse, and neutrophils were isolated using an EasySep mouse neutrophil enrichment kit (STEMCELL Tec, Vancouver, Canada). The isolated neutrophils were attached to slides. The prepared slides were incubated with 10-fold diluted serum from BWF1 mice treated with or without R848 for 30 min at 37°C. Subsequently, bound antibodies were detected using Alexa Fluor 488-conjugated donkey anti-mouse IgG (1:200; Invitrogen, Carlsbad, CA) and observed under a fluorescence microscope (BZ-X800; Keyence, Osaka, Japan).

### 2.3 Flow cytometry

Platelet-associated IgG (PAIgG) was measured using whole blood anticoagulated with ACD-A solution (2.2 w/v% sodium citrate hydrate, 0.8 w/v% citric acid hydrate, and 2.2 w/v% glucose). Samples for staining were prepared by removing erythrocytes from whole blood using Lysing Buffer (BD Biosciences, San Jose, CA). These samples were stained with predetermined optimal concentrations of antibodies (**Supplementary Table 1**) for 20 min at 4°C, washed in FACS buffer (PBS containing 0.1% BSA and 0.09% sodium azide), and then stained with optimal concentrations of appropriate fluorochrome-conjugated streptavidin for 20 min at 4°C. Cells were analyzed using FACSCalibur and LSRFortessa X-20 flow cytometers (BD Biosciences), and data were analyzed using FlowJo software (V10.8.1, BD Biosciences).

### 2.4 Histopathological analysis

Murine ileum, kidney, and liver tissues were fixed in 20% formalin and embedded in paraffin. The tissues were sectioned and stained as follows: the ileum (3-µm thickness) with hematoxylin-eosin (HE), Elastica van Gieson (EVG), and Victoria blue-HE (VB-HE); the liver (3-µm thickness) with HE and Elastica Masson stain; and the kidney (2-µm thickness) with HE and periodic acid-Schiff (PAS). The Swiss roll technique for intestinal pathology was performed based on a previously described (15). Histological staining reagents were obtained from MUTO PURE CHEMICALS (Tokyo, Japan).

Renal tissue injury was evaluated using the modified National Institutes of Health lupus nephritis activity and chronicity indices (16). All histological evaluations were performed by three independent investigators, at least two of whom were blinded to group allocation.

For immunofluorescence staining, the ileum and kidneys were embedded in OCT Compound (Sakura Finetek Japan, Tokyo, Japan), snap-frozen, sectioned at a 3 µm or 10 µm thickness and fixed in 4% paraformaldehyde. The ileum sections were immunostained with the antibodies listed in **Supplementary Table 1**. Histopathological specimens were observed using fluorescence and optical microscopy (BZ-X800; Keyence). Locally infiltrating cells were counted in up to three high-power fields (×200 magnification) per section by three investigators, and the mean number of cells per field was calculated for each animal. When evaluable fields were limited due to insufficient pathological section quality, the analysis was performed using the average of the available fields of view.

### 2.5 Intestinal flora and fecal metabolite analyses

Fecal samples from mice in each group were pooled prior to extraction. Total DNA was extracted from these pooled samples using a NucleoSpin DNA stool kit (TaKaRa, Kusatsu, Japan) according to the manufacturer’s instructions. Intestinal microbiota metagenomic analysis was performed by the Primary Cell Division of Cosmo Bio., Ltd. (Sapporo, Japan) using 16S rRNA gene amplicon sequencing on an Illumina MiSeq platform. The obtained raw paired-end reads data were merged using FLASH (Fast Length Adjustment of Short reads) software to combine paired-end reads. The assembled sequences were then subjected to noise reduction using CD-HIT-OTU/rDNATools, and bacterial species comprising the clustered OTUs (Operational Taxonomic Units) were identified and compared.

Fecal metabolite analysis was performed by TechnoSuruga Laboratory Co., Ltd. (Shizuoka, Japan) using gas chromatography with a flame ionization detector (GC-FID, 7890B, Agilent Technologies, Santa Clara, CA, USA) or high-performance liquid chromatography (HPLC, Organic acid analysis system, Shimadzu, Kyoto, Japan). The following compounds were measured: 1) short-chain fatty acids (acetic acid, propionic acid, n-butyric acid, isobutyric acid, n-valeric acid, isovaleric acid, and n-caproic acid) and 2) organic acids (succinic acid, lactic acid, and formic acid). For GC-FID measurement, 100 mg of each fecal sample was placed in a bead tube, and mixed with nine-fold volume of 0.5% phosphoric acid solution, and heat treatment at 85°C for 15 min. Next, the samples were homogenized with beads and centrifuged. An equal volume of ethyl acetate was added to the supernatant and mixed. Subsequently, an internal standard was added to the ethyl acetate layer recovered by centrifugation, and the target metabolites were quantified using GC-FID. For HPLC measurements, fecal samples were placed in bead tubes, extraction reagents were added, and heat-treated at 85°C for 15 min. The homogenized samples were centrifuged, and the supernatants were filtered through a 0.2 µm membrane filter. The filtrates were then quantitatively analyzed by HPLC using 5 mmol/L p-toluenesulfonic acid as the eluent, and 5 mmol/L p-toluenesulfonic acid, 100 µmol/L EDTA, and 20 mmol/L Bis-Tris as the reaction mixture. Because the measurement methods differed, the data were normalized prior to comparison. Fecal samples for these analyses were collected after 3 weeks of R848 treatment. The results were compared between the R848 (−) and R848 (+) groups.

### 2.6 Cells

Human umbilical vein endothelial cells (HUVEC) were obtained from Lonza (Basel, Switzerland), and human aortic smooth muscle cells (HAoSMC) were obtained from PromoCell (Heidelberg, Germany). HUVEC were maintained in EGM™-2 BulletKit (Lonza), and HAoSMC were maintained in Smooth Muscle Cell Basal Medium 2 (PromoCell).

### 2.7 *In vitro* cell assays

HUVEC were stimulated for 6 hours or 24 hours with IL-1β (10 ng/mL; R&D Systems, Minneapolis, MN, USA), TNF-α (10 ng/mL; R&D Systems), lipopolysaccharide (LPS; 0.5 µg/mL; *Escherichia coli* O111:B4; Sigma-Aldrich Japan), sodium propionate (1.5 mM; Sigma-Aldrich Japan), or trichostatin A (TSA; 1 nM; FUJIFILM Wako) alone, or in combination with an inflammatory stimulus (IL-1β, TNF-α, or LPS) and either sodium propionate or TSA. The cells were then subjected to quantitative polymerase chain reaction (qPCR) or western blotting. The assay medium was EBM-2 (Lonza) supplemented with 0.2% fetal bovine serum (FBS).

HAoSMC were treated with or without sodium propionate (1.5 mM) or sodium butyrate (1.5 mM; FUJIFILM Wako) for 48 hours or 7 days, and were then subjected to western blotting. The assay medium was RPMI-1640 (Sigma-Aldrich Japan, Tokyo, Japan) supplemented with 1% FBS.

### 2.8 RNA extraction and quantitative polymerase chain reaction

Total RNA was extracted from mouse ileum, kidney, and human cell lines, and reverse transcription-qPCR was performed as previously described (12). Briefly, total RNA was extracted using ISOGEN II (Nippon Gene, Tokyo, Japan) or an RNeasy Mini Kit (Qiagen, Hilden, Germany) according to the manufacturers instructions. The extracted RNA was reverse transcribed with PrimeScript RT Reagent Kit (TaKaRa). qPCR was performed using TB Green Premix Ex Taq (TaKaRa) on a QuantStudio 5 or QuantStudio 6 (Thermo Fisher Scientific). The data were quantified using standard curves and evaluated relative to the expression levels of *Gapdh* or *Actb* (mouse) and *ACTB* (human). In some experiments, the data were normalized to *Gapdh* (mouse) and calculated using the comparative Ct (ΔΔCt) method. Specific primer sequences are listed in **Supplementary Table 2**.

### 2.9 Western blotting

HUVEC and HAoSMC were lysed with RIPA buffer (BioDynamics Laboratory Inc., Tokyo, Japan), and SDS-PAGE and western blotting were performed as previously described (17). The antibodies used for western blotting were listed in **Supplementary Table 1**. Densitometric analysis was performed using ImageJ software (Rasband, W.S., ImageJ, U.S. National Institutes of Health, Bethesda, MD, USA).

### 2.10 Statistical analysis

Statistical analyses were performed using GraphPad Prism 10 software (GraphPad Software, La Jolla, CA, USA). In the animal study, differences between groups were compared using Student’s *t*-test (parametric test for two groups), Mann-Whitney *U* test (nonparametric test for two groups), a one-way ANOVA followed by Sidak’s multiple-comparison test (for multiple groups), and Fisher’s exact test (for fecal occult blood testing). In the cell culture studies, differences between groups were compared using a one-way ANOVA followed by Sidak’s multiple-comparison test (for multiple groups).

## 3 Results

### 3.1 Continuous administration of R848 to young female mice induced intestinal lesions

We continuously administered R848 to BWF1 mice and observed the resulting phenotype. Repeated treatment with R848 significantly induced bloody stools in the treatment group from 3 to 6 weeks post-treatment. Fecal occult blood tests became positive at 3 weeks after R848 treatment (**Table 1**). At 6 weeks post-treatment, macroscopic bleeding was observed in the small intestine (**Supplementary Figure 1A**). Based on these symptoms, we considered the possibility of LMV. Because lesions in LMV often appear from the jejunum to the ileum (4), we prepared and observed Swiss roll preparations of the small intestine. HE staining of Swiss roll preparations demonstrated intestinal wall thickening from the jejunum to the ileum (**Supplementary Figure 1B**). In particular, significant bleeding was observed in the ileum (**Supplementary Figure 1C**). Therefore, we focused our subsequent analyses on the ileum.

**Table 1:** Continuous stimulation with R848 induced fecal occult blood positivity in NZBWF1 (BWF1) mice.

LMV induction time	Positive frequency (Positive/Total)		*P* value (Fisher’s exact test)
	R848(−)	R848(+)	R848(−) vs. R848(+)
0 weeks	11.1% (2/18)	0% (0/18)	ns
1 weeks	11.1% (2/18)	11.1% (2/18)	ns
2 weeks	0% (0/18)	11.1% (2/18)	ns
3 weeks	0% (0/18)	66.6% (12/18)	< 0.0001
4 weeks	0% (0/18)	100% (18/18)	< 0.0001
5 weeks	0% (0/18)	94.1% (16/17)	< 0.0001
6 weeks	0% (0/18)	94.1% (16/17)	< 0.0001

R848 was applied to one ear of eight-weeks-old female BWF1 mice three times a week for 6 weeks. Fecal occult blood was assessed at the indicated time points. LMV, lupus mesenteric vasculitis, ns, not significant.

**Figure 1:**
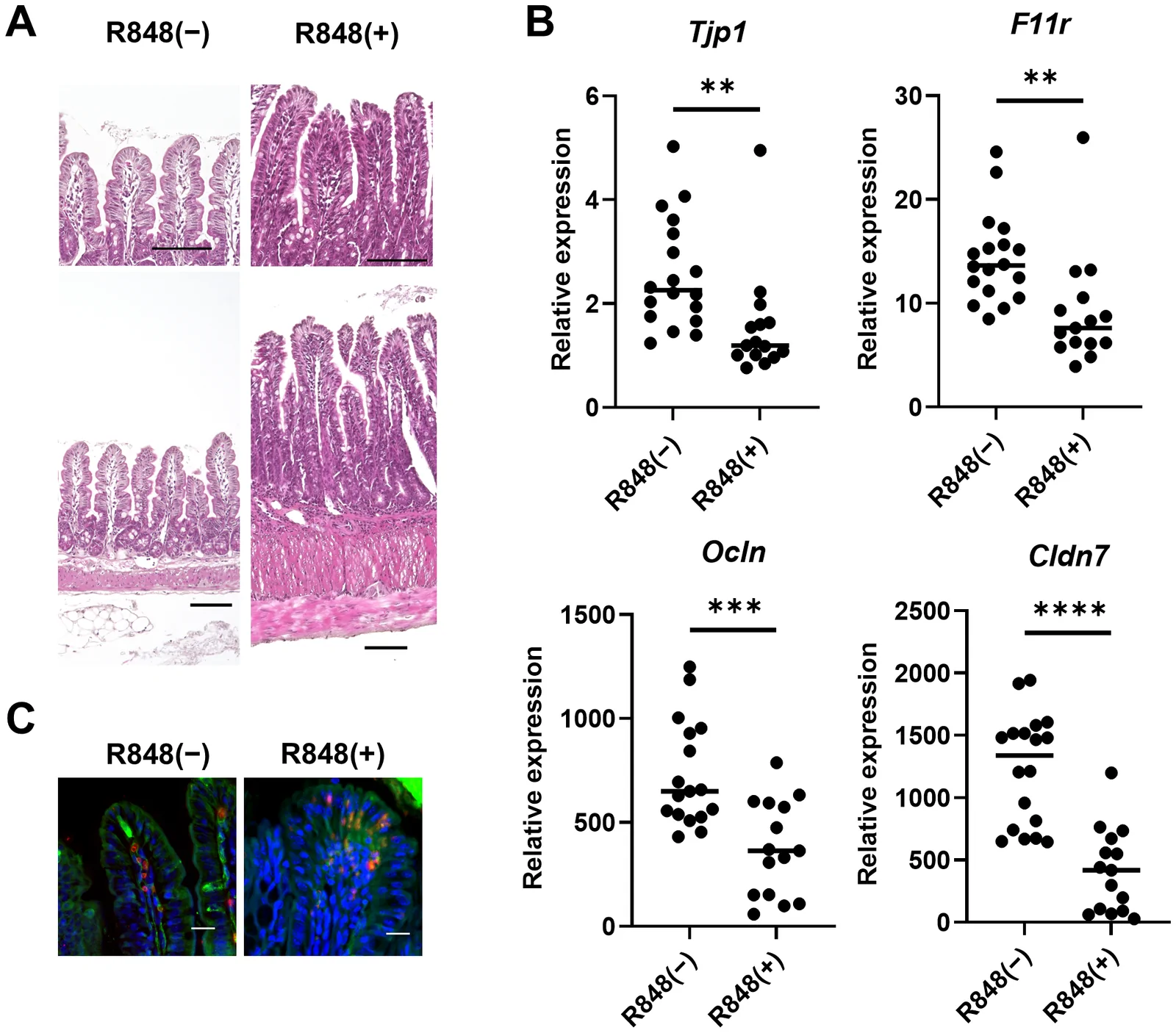
Continuous R848 stimulation induces intestinal bleeding in NZBWF1 (BWF1) mice. Eight-week-old female BWF1 mice were treated with R848 three times weekly on one ear for 6 weeks. **(A)** Representative images of ileum sections from BWF1 mice stained with the hematoxylin-eosin (HE). Scale bar = 100 µm. **(B)** A portion of the ileum was collected from BWF1 mice, and total RNA was extracted. The expression levels of *Tjp1*, *F11r*, *Ocln*, and *Cldn7* were determined by quantitative polymerase chain reaction (qPCR). The expression of the indicated genes was normalized to that of *Gapdh*. Each dot represents an individual mouse (R848(−)*n* = 18, R848(+)*n* = 15). Statistical analysis was performed using a two-tailed Student’s *t*-test. Asterisks indicate statistically significant differences (**p < 0.01, ***p < 0.001, ****p < 0.0001). **(C)** Immunofluorescent staining for red blood cells (red) and CD31 (capillary vascular endothelium; green) was performed on a paraffin-embedded ileum sections. Nuclei were counterstained with DAPI (blue). Scale bar = 20 µm.

Histopathological analysis of ileum sections using HE staining revealed thickening of the intestinal wall, cellular infiltration, and damage to the intestinal epithelium in R848-treated mice (**Figure 1A**). To confirm R848-induced epithelial injury, we measured the gene expression of tight junction components, including *Tjp1*, *F11r*, *Ocln*, and *Cldn7*, in the ileum by qPCR. The expression levels of these genes were decreased in the ileum of R848-treated mice (**Figure 1B**). These results suggest that the permeability of the intestinal epithelium may have increased. To examine bleeding histologically, we performed immunofluorescent staining of red blood cells and capillary endothelium (CD31). In R848-treated mice, disruption of vascular endothelial alignment was observed, with erythrocytes detected outside the vessels (**Figure 1C**).

### 3.2 Treatment with R848 caused earlier onset of SLE symptoms

**Figure 2:**
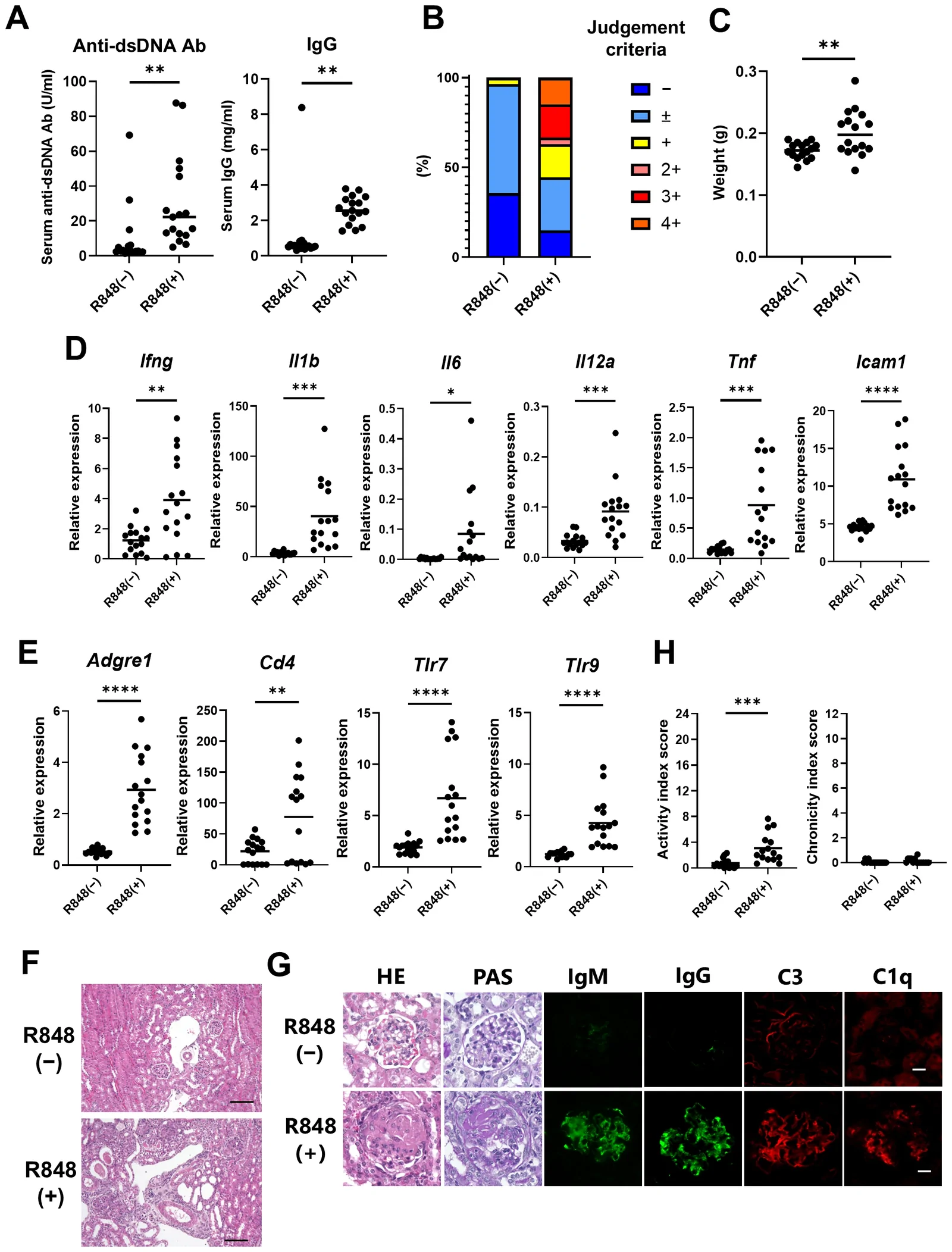
R848 stimulation leads to the development of lupus nephritis in BWF1 mice. Eight-week-old female BWF1 mice were treated with R848 for 6 weeks. **(A)** Serum was collected after 6 weeks of R848 treatment, and the anti-dsDNA antibody levels were measured by ELISA (R848(−)*n* = 18, R848(+)*n* = 17). **(B)** Proteinuria was evaluated using dipsticks at the time of sampling in BWF1 mice treated with or without R848 (R848(−)*n* = 28, R848(+)*n* = 27). The ratio of each score is shown. **(C)** Kidney weights were measured in BWF1 mice treated with or without R848 for 6 weeks (R848(−)*n* = 16, R848(+)*n* = 16). **(D)** Whole kidneys were harvested from these mice, and RNA was extracted. The expression levels of *Ifng*, *Il1b*, *Il6*, *Il12a*, *Tnf*, and *Icam1* were determined by quantitative polymerase chain reaction (qPCR). The expression levels of the indicated genes were normalized to that of *Actb* (R848(−)*n* = 16, R848(+)*n* = 16). **(E)** Whole kidneys were harvested from these mice, and RNA was extracted. The expression levels of *Adgre1*, *Cd4*, *Tlr7*, and *Tlr9* were determined by qPCR. The expression levels of the indicated genes were normalized to that of *Actb* (R848(−)*n* = 16, R848(+)*n* = 16). **(F)** Representative images of kidney sections from these mice stained with hematoxylin-eosin (HE). Scale bar = 100 µm. **(G)** Representative images of kidney sections stained with HE, periodic acid-Schiff (PAS), anti-IgM, anti-IgG, anti-C3, and anti-C1q. Scale bar = 20 µm. **(H)** The kidney pathological score (modified National Institutes of Health (NIH) activity and chronicity index). Statistical analysis was performed using a two-tailed Student’s *t*-test **(A, C, D, E)** and the nonparametric Mann-Whitney *U* test **(H)**. Each dot represents an individual mouse. Asterisks indicate statistically significant differences (*p < 0.05, **p < 0.01, ***p < 0.001, ****p < 0.0001).

Although female BWF1 mice are a well-known model for SLE, their autoantibody levels typically increase only as they age (18). As described above, we demonstrated that continuous administration of R848 to young female BWF1 mice caused intestinal damage. Therefore, we investigated whether autoimmunity is involved in these symptoms. First, we measured the serum levels of anti-dsDNA antibodies and IgG 6 weeks after R848 treatment (at 14 weeks of age), and found that both were significantly increased in the R848-treated mice (**Figure 2A**). The R848-treated mice also exhibited an increased frequency of proteinuria (≥2+) development (**Figure 2B**). After 6 weeks of R848 treatment, their kidney weights increased significantly (**Figure 2C**). Additionally, a qPCR analysis of the whole kidney showed that the expressions of inflammatory genes (**Figure 2D**) and surface markers of inflammatory cells (**Figure 2E**) were increased in the R848-treated mice. Pathological evaluation of the R848-treated mice showed accelerated interstitial inflammation (**Figure 2F**), glomerular lesions, and deposition of immune complexes in glomeruli (**Figure 2G**). In addition, evaluation of renal tissue injury using the modified National Institutes of Health lupus nephritis activity and chronicity indices revealed a significant increase in the pathological score, particularly in the activity index (**Figure 2H**). In contrast, IMQ treatment of young BWF1 mice did not change their symptoms (12). These results suggest that the continuous administration of R848 to young mice can trigger SLE pathology early. Therefore, the promoted autoimmunity may contribute to intestinal damage.

### 3.3 Continuous administration of R848 to young female mice induced LMV

**Figure 3:**
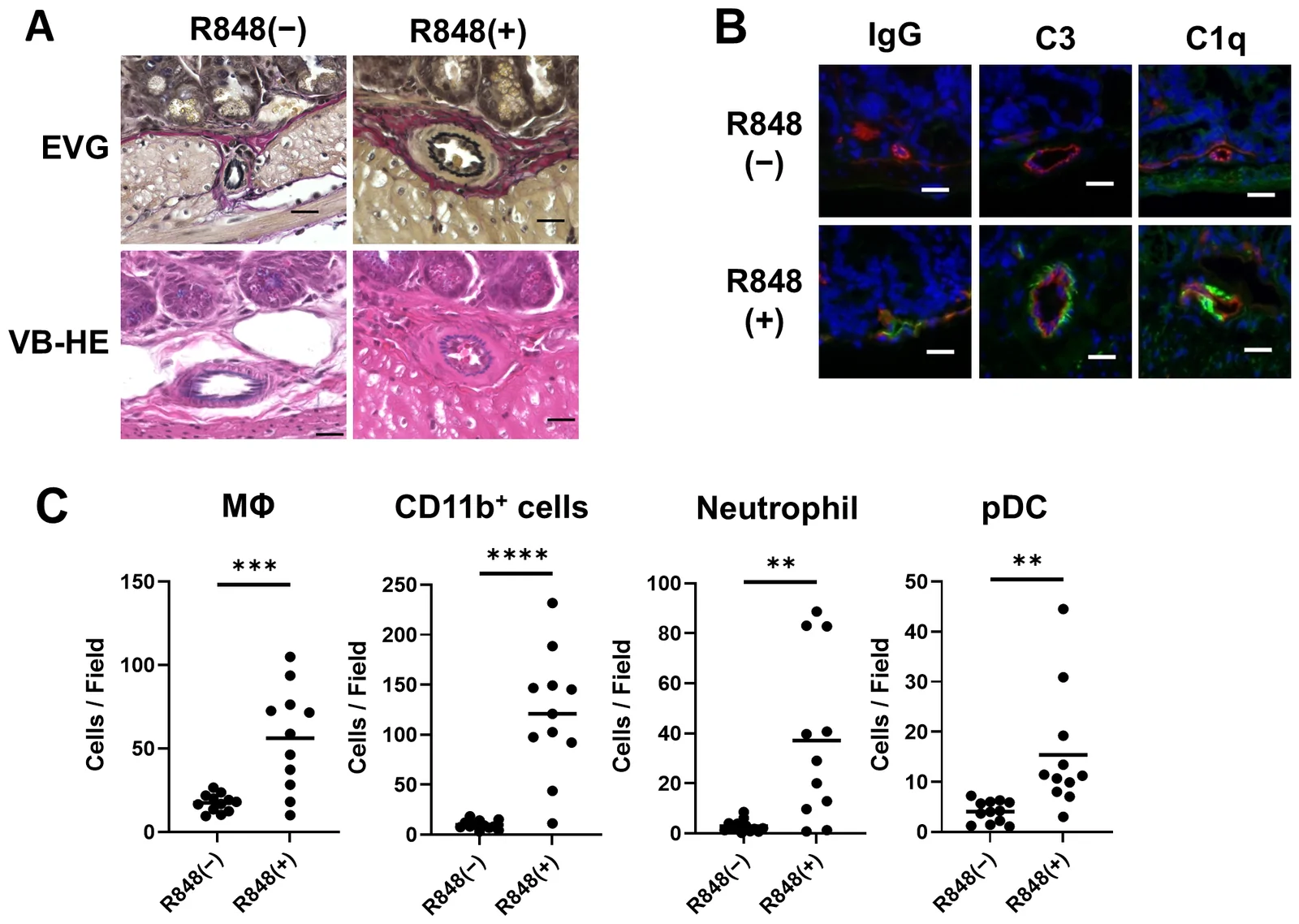
Autoimmune vasculitis is induced in the intestines of R848-stimulated BWF1 mice. Eight-week-old female BWF1 mice were treated with R848 for 6 weeks. **(A)** Representative images of blood vessels in ileum sections from BWF1 mice stained with Elastica-Van Gieson (EVG) and Victoria blue HE (VE-HE). Scale bar = 20 µm. **(B)** To detect immune complex deposition on the ileal vessels, sections were co-stained with anti-CD31 (vascular endothelium: red), anti-IgG, anti-C3 or anti-C1q (green), and DAPI (blue). Scale bar = 20 µm. **(C)** Inflammatory cell infiltration was analyzed using the following markers: F4/80 [macrophages (MΦ)]; CD11b (granulocytes, monocytes, macrophages, part of B cells, etc.); myeloperoxidase and Ly6G (neutrophils); and PDCA-1 and CD11c (plasmacytoid dendritic cells (pDC)). The numbers of MΦ, CD11b^+^ cells, neutrophils, and pDC were counted in up to three high-power fields (×200 magnification) per section. Each dot represents an individual mouse (R848(−): *n* = 12, R848(+): *n* = 11). Statistical analysis was performed using a two-tailed Student’s *t*-test. Asterisks indicate statistically significant differences (**p < 0.01, ***p < 0.001, ****p < 0.0001).

As shown in **Figure 1**, R848-treated mice exhibited a high incidence of intestinal bleeding, suggesting not only epithelial but also vascular abnormalities. To address this, we analyzed the blood vessels in the ileum histologically using Elastica-van Gieson (EVG) staining and Victoria blue (VB)-HE staining. In R848-treated mice, the ileal blood vessels exhibited marked swelling of vascular endothelial cells, thickening of the blood vessel wall, and perivascular infiltration of inflammatory cells (**Figure 3A**). These findings suggested the onset of vasculitis. Since SLE symptoms were also established at this point, we investigated whether immune complexes were deposited in the blood vessels of R848-treated mice. Immunofluorescent microscopy analysis demonstrated the deposition of immune complexes, including IgG, C3, and/or C1q complement, in the blood vessels of the ileum in R848-treated mice (**Figure 3B**). In addition, anti-neutrophil cytoplasmic antibodies (ANCA) associated with vasculitis were detected in the sera from R848-treated mice (**Supplementary Figure 2**). Next, we evaluated inflammatory cells that infiltrated intestinal lamina propria and submucosa. We assessed the presence of various leukocyte populations, including macrophages (MΦ; F4/80^+^), CD11b^+^ cells (such as granulocytes, monocytes macrophages, and some B cells), neutrophils (MPO and Ly6G double-positive), and plasmacytoid dendritic cells (pDC; PDCA-1 and CD11c double-positive), by immunostaining, and counted the number of intestinal lamina propria and submucosa. The infiltration of these inflammatory cells was significantly increased in the intestinal lamina propria and submucosa of R848-treated mice (**Figure 3C**). Taken together, our data demonstrated that the continuous administration of R848 to young female BWF1 mice induced autoimmune vasculitis in the ileum as well as SLE pathology. Thus, these mice developed LMV pathology. Furthermore, these symptoms were similar to those of human LMV pathology (5), indicating that we have established a new protocol for generating an LMV mouse model.

We then investigated whether similar tissue changes occur at an earlier time point in BWF1 mice, and whether they also develop in other SLE model mice, such as BXSB and MRL/*lpr* mice. After 3 weeks of R848 treatment in BWF1 mice, ileal and vascular lesions were observed, similar to those seen after 6 weeks of treatment. In addition, comparable pathological changes were also observed in BXSB and MRL/*lpr* mice (**Supplementary Figures 3A, B**). In contrast, wild-type BALB/c mice showed no pathological changes after 6 weeks of R848 treatment (**Supplementary Figures 3C, D**). In addition, when IMQ cream was similarly applied to BWF1 mice three times a week for 6 weeks, no major pathological changes were observed (**Supplementary Figures 3E, F**). Therefore, these data suggest that LMV can be induced only when lupus-prone mice are treated consecutively with R848. Based on these results, we utilized this model as an induced LMV mouse model.

### 3.4 Intestinal microbiota are implicated in the exacerbation and onset of LMV lesions

**Figure 4:**
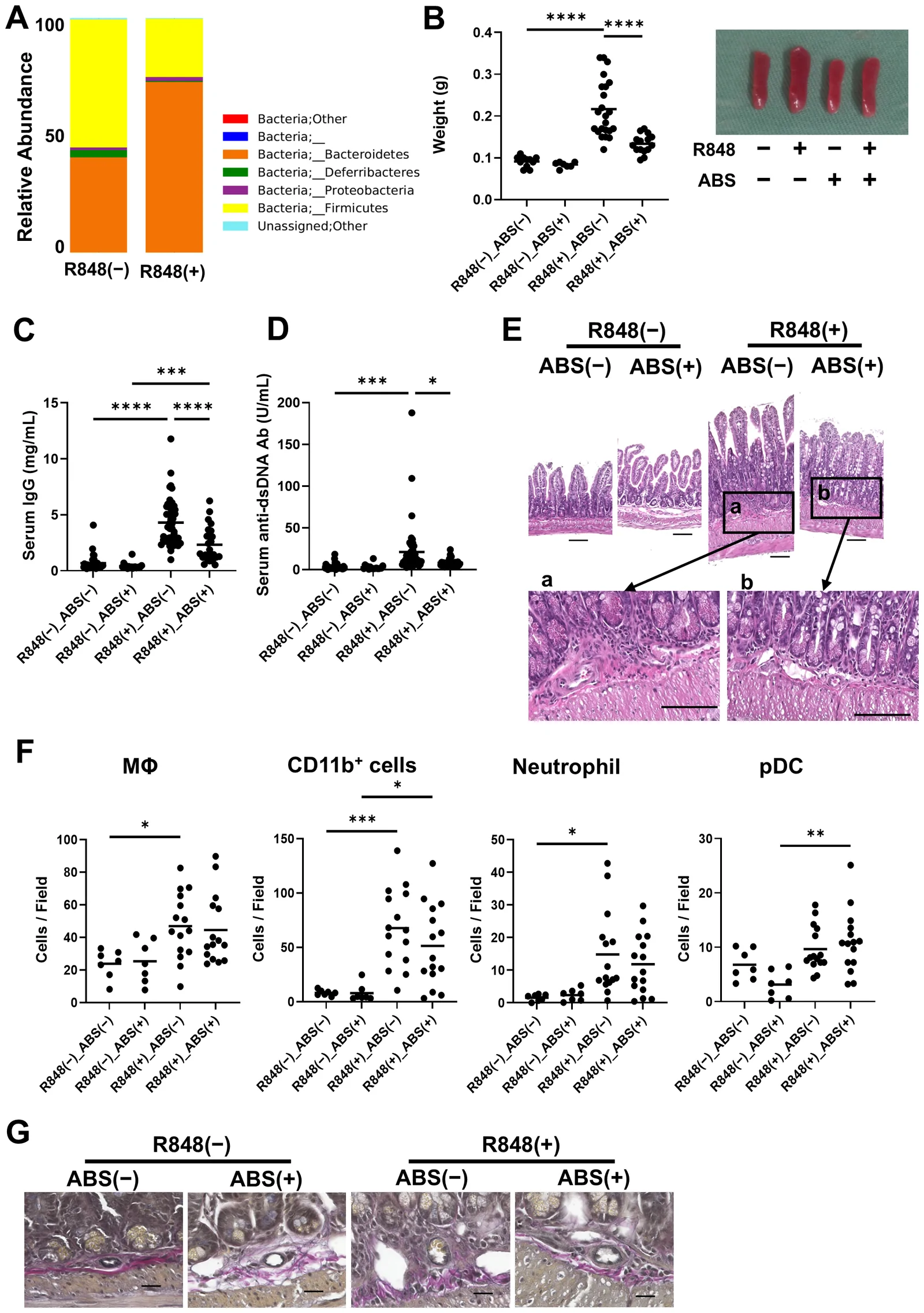
Intestinal microbiota was involved in the onset of LMV lesions in LMV-induced mice. Eight-week-old female BWF1 mice were treated with R848 three times weekly on one ear for 3‐6 weeks. **(A)** Mice were treated for 6 weeks. DNA was extracted from pooled fecal samples for each group and metagenomic analysis of the intestinal microbiota was performed using 16S rRNA amplicon sequencing. Bacterial composition at the phylum level is shown as relative abundance. **(B–G)** Mice were treated for 3 weeks with or without an antibiotic cocktail comprising vancomycin, neomycin, ampicillin, and metronidazole, which was administered from 3 weeks prior to and continuously during the R848 treatment. **(B)** Spleen weight (left) and representative photos of spleens (right) from BWF1 mice are shown. Spleen weightsR848(−)_ABS(−)*n* = 11, R848(−)_ABS(+)*n* = 7, R848(+)_ABS(−)*n* = 23, R848(+)_ABS(+)*n* = 15. **(C, D)** Serum was collected, and **(C)** total IgG and **(D)** anti-dsDNA antibody levels were measured by ELISA (R848(−)_ABS(−)*n* = 33, R848(−)_ABS(+)*n* = 17, R848(+)_ABS(−)*n* = 45, R848(+)_ABS(+)*n* = 25). **(E)** Representative photos of HE-stained ileum sections from BWF1 mice are shown. Scale bar = 100 µm. Infiltrating inflammatory cells were observed in the areas enclosed by squares. **(F)** Inflammatory cell infiltrates were analyzed using following markers: F4/80 (MΦ); CD11b (granulocytes, monocytes, macrophages, part of B cells, etc.); myeloperoxidase and Ly6G (neutrophils); and PDCA-1 and CD11c (pDC). The numbers of MΦ, CD11b^+^ cells, neutrophils, and pDC were counted up to three high-power fields (×200 magnification) per section (R848(−)_ABS (−)*n* = 7, R848(−)_ABS(+)*n* = 7, R848(+)_ABS(−)*n* = 15, R848(+)_ABS(+)*n* = 15). **(G)** Representative photos of EVG-stained ileum sections from the BWF1 mice are shown. Scale bar = 20 µm. Statistical analysis was performed using a one-way ANOVA followed by Sidak’s multiple comparison test **(B–D, F)**. Each dot represents an individual mouse. Asterisks indicate statistically significant differences (*p < 0.05, **p < 0.01, ***p < 0.001, ****p < 0.0001). ABS, antibiotics.

Next, we investigated the cause of LMV lesions induced by continuous administration of R848 to young female BWF1 mice. In recent years, intestinal microbiota changes have been implicated in the pathology of autoimmune diseases and inflammatory bowel diseases (19–21). Therefore, we extracted DNA from feces 6 weeks after R848 treatment and performed metagenomic analysis of the intestinal microbiota. In the LMV-induced mouse model (R848(+) group), the abundance of *Bacteroidetes* increased and that of *Firmicutes* decreased, indicating changes in the intestinal microbiota with continuous administration of R848 (**Figure 4A**). Furthermore, the LMV-induced mice showed a decrease in α-diversity (Shannon and Chao1 indices) compared with control mice. In addition, the principal coordinate analysis plot for β-diversity revealed that the two samples were plotted far apart, indicating that R848 stimulation substantially altered the overall microbial community structure (**Supplementary Figure 4**). As described above, R848 treatment induced bloody stools beginning at 3 weeks post-treatment (**Table 1**). To investigate the role of intestinal microbiota in this pathology, we administered broad-spectrum antibiotics, a combination of four antibiotics (vancomycin, neomycin, ampicillin, and metronidazole), to deplete the intestinal microbiota and evaluate LMV development. Consistent with previous findings, fecal occult blood became significantly positive after 3 weeks of R848 treatment, but this positivity was suppressed by antibiotic treatment. However, the suppressive effect was diminished after 6 weeks of R848 treatment (**Table 2**).

**Table 2:** Frequency of fecal occult blood positivity in LMV-induced mice with and without antibiotic treatment.

Group	A	B	C	D			
ABS	−	+	−	+			
R848	−	−	+	+			
LMV induction time	Positive frequency (Positive/Total)				*p* value (Fisher’s exact test)		
	A	B	C	D	A vs. C	B vs. D	C vs. D
0 weeks	0% (0/13)	0% (0/13)	0% (0/15)	0% (0/15)	ns	ns	ns
1 weeks	0% (0/17)	0% (0/17)	0% (0/25)	0% (0/25)	ns	ns	ns
2 weeks	0% (0/17)	0% (0/17)	4% (1/25)	8% (2/25)	ns	ns	ns
3 weeks	0% (0/17)	0% (0/17)	68% (17/25)	12% (3/25)	< 0.0001	ns	0.0001
4 weeks	0% (0/10)	0% (0/10)	90% (9/10)	60% (6/10)	0.0001	0.0108	ns
5 weeks	0% (0/10)	0% (0/10)	100% (10/10)	50% (5/10)	<0.0001	0.0325	0.0325
6 weeks	10% (1/10)	0% (0/10)	90% (9/10)	70% (7/10)	0.0011	0.0031	ns

R848 was applied to one ear of eight-week-old female BWF1 mice three times a week for 6 weeks. Three weeks prior to R848 treatment initiation, an antibiotic cocktail consisting of vancomycin (0.5 g/L), neomycin (1.0 g/L), ampicillin (1.0 g/L), and metronidazole (1.0 g/L) was administered in drinking water. During weeks 0 to 6, the mice were tested for fecal occult blood. LMV, lupus mesenteric vasculitis; ABS, antibiotics; ns, not significant.

Next, because LMV-induced mice exhibited chronic bloody stools, we focused on hemostatic function. SLE is known to induce blood abnormalities such as thrombocytopenia (22). Thus, we collected whole blood and measured platelet counts, which play a crucial role in hemostasis. A significant decrease in the platelet count was observed 3 weeks after R848 treatment; however, this count was normalized by administration of antibiotics (**Supplementary Figure 5A**). Furthermore, SLE patients produce platelet-associated IgG (PAIgG), an autoantibody that accelerates platelet destruction (22, 23). Given that LMV-induced mice develop SLE-like pathology, we measured PAIgG levels to investigate the cause of thrombocytopenia. We found that PAIgG levels were increased by LMV induction (**Supplementary Figure 4B**). These results showed that the decrease in platelet count in the LMV mouse model was due to platelet destruction caused by an increase in PAIgG. Interestingly, antibiotic administration reduced these elevated PAIgG levels to the normal range, demonstrating that the intestinal microbiota plays a crucial role in the increase in PAIgG (**Supplementary Figure 5B**). To elucidate how bacteria are involved in the exacerbation of LMV pathology, we subsequently focused on the pathology up to 3 weeks, as fecal occult blood was most significantly suppressed by antibiotic administration at this time point.

In recent years, the translocation of intestinal microbiota into the systemic circulation has been reported in SLE (24). To assess the contribution of intestinal microbiota to systemic immune activation in our model, we examined spleen weights. Splenomegaly was observed after R848 treatment; however, this enlargement was suppressed by antibiotic administration (**Figure 4B**). Next, we measured anti-dsDNA antibody and IgG levels in mouse serum. Serum anti-dsDNA antibody and IgG levels were increased in R848-treated BWF1 mice, and these increases were suppressed by the administration of antibiotics (**Figures 4C, D**). These results demonstrated that intestinal microbiota promote autoimmunity.

We examined the effects of antibiotic administration on ileal pathology induced by R848 treatment. In the pathological analysis of the ileum using HE staining at 3 weeks post-R848 treatment, no significant changes were observed in the intestinal epithelium or intestinal wall thickening following antibiotic administration, although cellular infiltration in the submucosal and lamina propria tended to be slightly suppressed (**Figure 4E**). However, despite this qualitative observation, quantitative analysis revealed that the increased numbers of infiltrating cells –MΦ, CD11b^+^ cells, and neutrophils– induced by R848 treatment remained unchanged even after antibiotic administration (**Figure 4F**). Next, since vasculitis was observed with continued R848 treatment (**Figure 3A**), we evaluated the effects of intestinal microbiota on vasculitis in the blood vessels of the intestinal submucosal lamina propria using EVG staining. R848-induced vasculitis tended to be suppressed by antibiotic administration, as indicated particularly by attenuated vascular endothelial swelling (**Figure 4G**). These findings suggest that intestinal microbiota may be involved in the pathogenesis of LMV.

Since antibiotic-treated mice also exhibited increased fecal occult blood positivity during the late phase of R848 administration, we examined the pathological changes associated with LMV and SLE after 6 weeks of R848 treatment. In contrast to the significant reduction at 3 weeks post-R848 treatment, splenomegaly tended to be reduced by antibiotic administration (**Supplementary Figure 6A**). In addition, serum IgG and anti-dsDNA antibody levels were not significantly decreased (**Supplementary Figures 6B, C**). Ileal histology and vasculitis pathology exhibited similar trends to those observed at 3 weeks (**Supplementary Figures 6D, E**). However, immunofluorescence microscopy analysis demonstrated that although IgG deposition was reduced, the deposition of immune complexes, including C3 and/or C1q complements, was still observed within the blood vessels of the ileum in LMV-induced mice (**Supplementary Figure 6F**). These results suggest that the intestinal microbiota may play a role in the early stages of LMV pathogenesis.

### 3.5 Vascular inflammation observed in LMV pathology is influenced by bacteria

**Figure 5:**
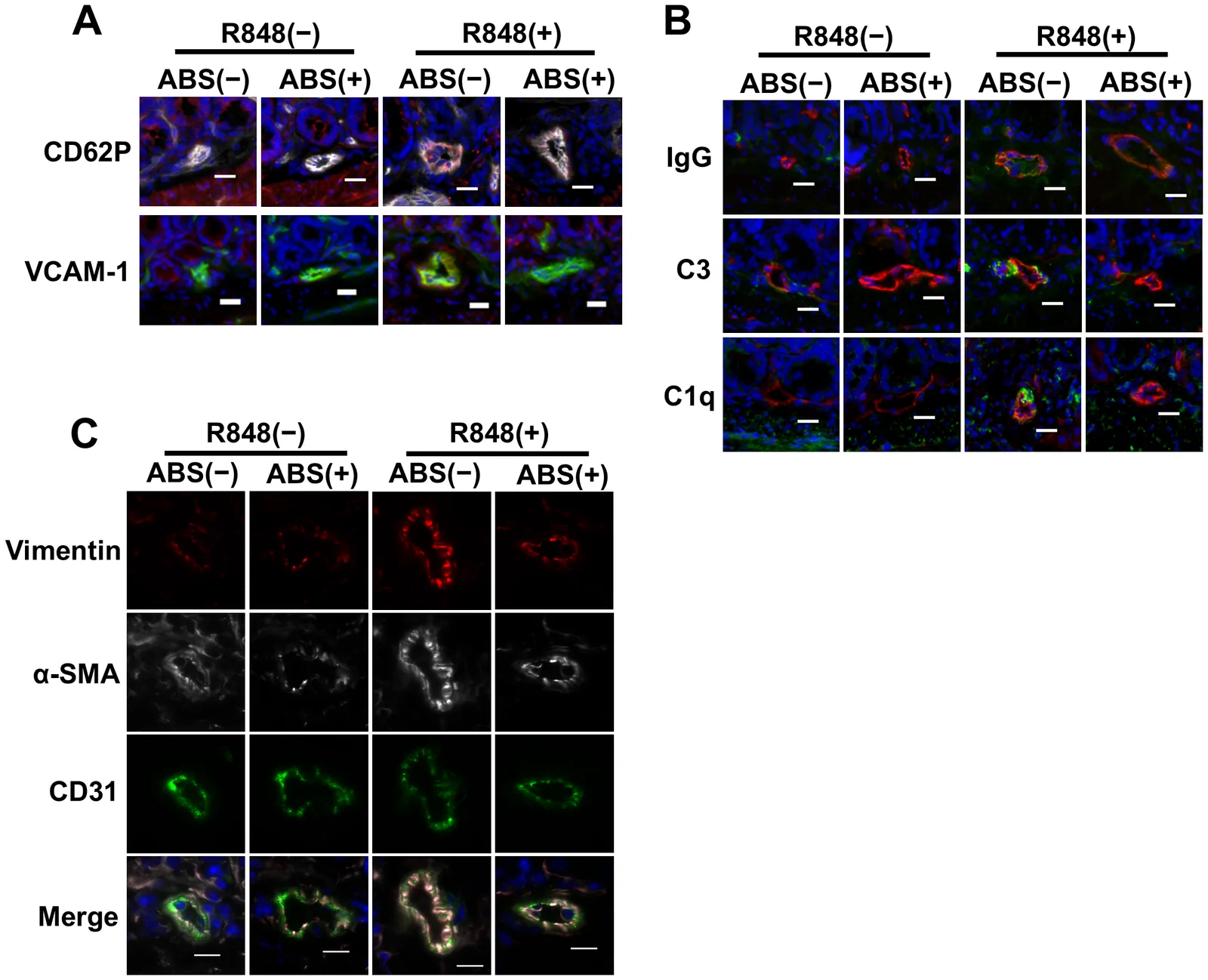
Vascular inflammation observed in LMV pathology is influenced by bacteria. Eight-week-old female BWF1 mice were treated with R848 three times per week on one ear for 3 weeks. An antibiotic cocktail comprising vancomycin, neomycin, ampicillin, and metronidazole was administered 3 weeks prior to and continuously during the R848 treatment. **(A–C)** Representative images of ileum sections from BWF1 mice. Sections were stained for [**(A)** upper panel] CD62P (P-selectin; red) and CD31 (white), and [**(A)** lower panel] VCAM-1 (red) and CD31 (green); **(B)** IgG, C3, or C1q (green) and CD31 (red); and **(C)** vimentin (red), α-smooth muscle actin (α-SMA; white), and CD31 (green). Nuclei were counterstained with DAPI (blue). Scale bars = 20 µm **(A, B)** and 10 µm **(C)**.

Next, we investigated vascular inflammation and its association with the intestinal microbiota, specifically focusing on the activation of vascular endothelial cells and the dedifferentiation of vascular smooth muscle cells (VSMC). Consistent with a previous report that LMV often induces inflammation in the blood vessels of the small intestine (25), LMV-induced mice in our study showed thickening of the small blood vessel walls in the small intestine (**Figure 3A**). Vascular inflammation in cardiovascular diseases involves activated vascular endothelial cells and dedifferentiated VSMC (26). However, it remains unclear whether a similar cellular mechanism underlies the vascular inflammation observed in LMV pathology. To address this, we investigated whether the swelling of vascular endothelial cells and the thickening of the vascular wall observed upon LMV induction were also caused by the activation of vascular endothelial cells and the dedifferentiation of VSMC. Thus, we examined vascular endothelial cells and VSMC 3 weeks post-R848 treatment, with or without antibiotic administration. To confirm endothelial cell activation, we examined the expression of CD62P and VCAM-1 using immunofluorescence staining. The expression of both markers was increased in LMV-induced mice, and this vascular endothelial cell activation was suppressed by antibiotic administration (**Figure 5A**). Unlike the late phase, the deposition of immune complexes in vascular endothelial cells observed upon LMV induction was also suppressed by antibiotic administration at this early stage (**Figure 5B**). Thus, autoimmune vasculitis was also suppressed by antibiotic administration. Furthermore, vimentin expression was markedly increased in the VSMC of LMV-induced mice, indicating a trend of dedifferentiation toward a synthetic phenotype. However, antibiotic administration inhibited this dedifferentiation (**Figure 5C**). Thus, these data indicate that the intestinal microbiota plays a crucial role in the phenotypic changes of VSMC and in the activation of vascular endothelial cells.

### 3.6 Intestinal microbiota contributes to the exacerbation of LMV pathology

Previous reports have suggested that the translocation of intestinal microbiota into the systemic circulation may promote autoimmune diseases such as SLE (24). In this study, we found that the expression of tight junction-related genes was decreased by R848 treatment (**Figure 1B**), and that the R848-induced reduction of *Cldn7* expression was unaffected by antibiotic administration (**Supplementary Figure 7A**). We next focused on *Lypd8*; although it is primarily known to play a crucial role in preventing intestinal bacterial translocation in the large intestine, it is also expressed in the small intestine (27). Likewise, *Lypd8* gene expression was decreased following R848 treatment and remained suppressed even after antibiotic administration (**Supplementary Figure 7B**). In addition, we observed an increase in liver weight following R848 treatment, but antibiotic administration prevented this increase (**Supplementary Figure 7C**). Thus, these results suggest that intestinal barrier dysfunction may contribute to systemic immune activation. Next, we investigated liver pathology using HE and EM staining; however, we observed no notable changes (**Supplementary Figure 7D**), suggesting that liver abnormalities had limited involvement in the pathology of LMV or the increased intestinal bleeding. These findings suggest that systemic bacterial components resulting from barrier dysfunction may not only promote SLE pathology but also exert adverse effects on blood vessels, thereby contributing to the exacerbation of LMV pathology.

### 3.7 Propionate suppressed endothelial cell activation and dedifferentiation of vascular smooth muscle cells

**Figure 6:**
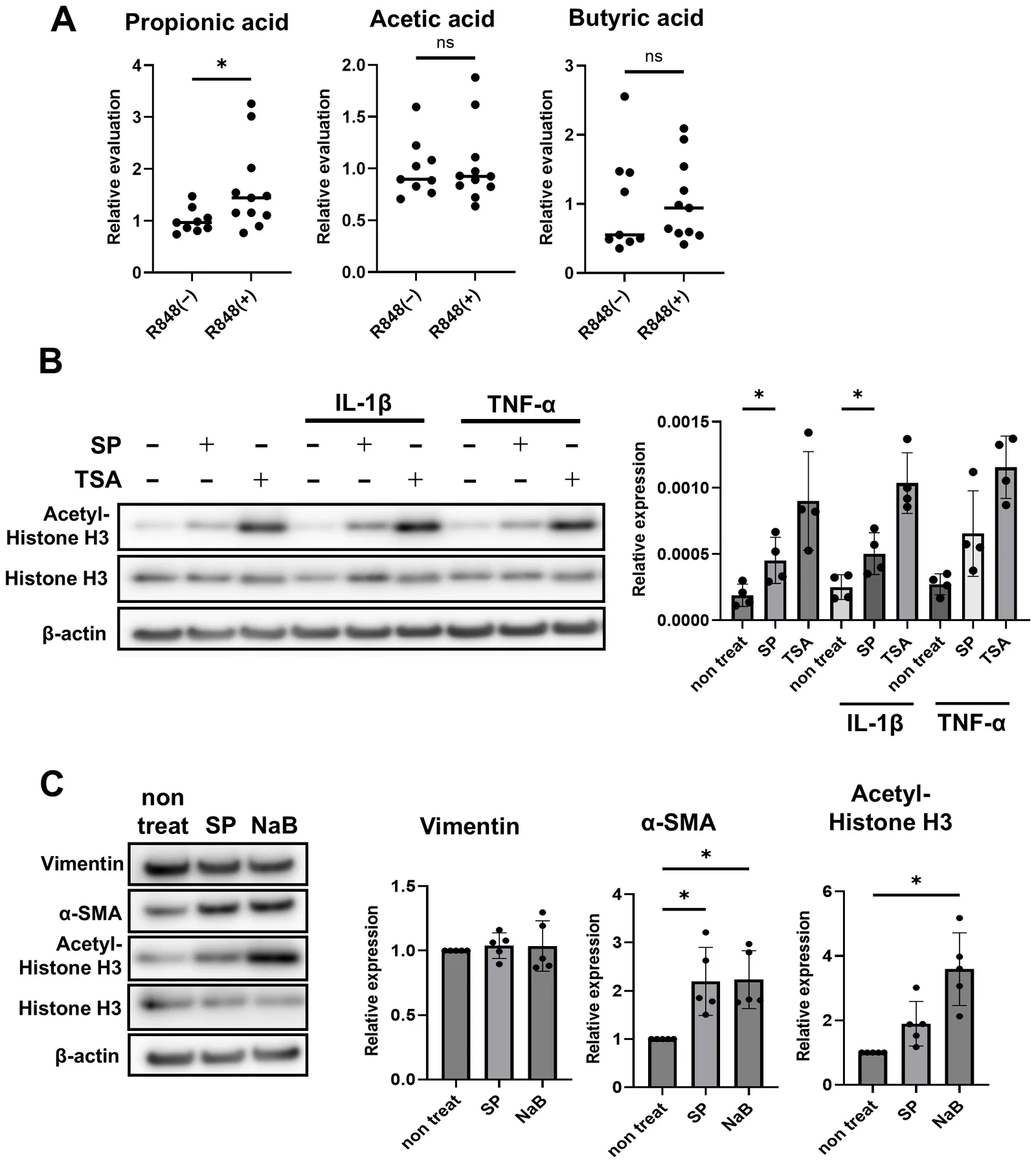
Effects and mechanisms of propionate on vascular cells. **(A)** Eight-week-old female BWF1 mice were treated topically with R848 on one ear three times per week for 3 weeks. Fecal metabolite analysis was performed on samples collected 3 weeks after R848 treatment to measure the concentrations of propionic acid, acetic acid, and butyric acid. Because the measurement methods differed, the data were normalized before comparison (R848(−): *n* = 9, R848(+): *n* = 11). **(B)** HUVEC were treated with or without IL-1β or TNF-α in the presence or absence of sodium propionate (SP) or trichostatin A (TSA) for 6 h, and protein expression was examined by western blotting. The left panel shows representative bands for acetyl-histone H3 (Lys9), histone H3, and β-actin. The right graph shows the results of densitometric analysis of the bands. Protein levels were normalized to those of histone H3 and β-actin (*n* = 4). **(C)** Human aortic smooth muscle cells (HAoSMC) were exposed to SP and sodium butyrate (NaB) for 48 h, and protein expression was examined by western blotting. The left panel shows representative protein bands for vimentin, α-SMA, acetyl-histone H3 (Lys9), histone H3, and β-actin. The right graph shows the results of densitometric analysis of the bands. The expression levels of vimentin and α-SMA were normalized to that of β-actin, and the expression level of acetyl-histone H3 (Lys9) was normalized to those of histone H3 and β-actin (*n* = 5). Statistical analyses were performed using the nonparametric Mann-Whitney *U* test **(A)** and one-way ANOVA followed by Sidak’s multiple comparison test **(B, C)**. Each dot represents an individual mouse or experiment. Asterisks indicate statistically significant differences (ns; not significant, *p < 0.05).

Our results suggest that the intestinal microbiota may be involved in the progression of LMV pathology. To further elucidate their impact, we focused on the metabolites of intestinal bacteria, which are key mediators in host-microbiota interactions. First, we performed a targeted metabolomic analysis of fecal samples from BWF1 mice 3 weeks after LMV induction to compare short-chain fatty acid profiles. This analysis demonstrated that propionic acid levels increased in feces 3 weeks after LMV induction. In contrast, the levels of other major short-chain fatty acids, acetic acid and butyric acid, did not change significantly following LMV induction (**Figure 6A**).

Next, to explore the physiological significance of the LMV-induced increase in propionic acid levels within the intestinal tract, we investigated the effects of propionate on vascular endothelial and vascular smooth muscle, which constitute the vasculature. To mimic the inflammatory microenvironment observed in LMV pathology, HUVEC were stimulated with IL-1β and TNF-α, representing cytokines secreted by perivascular infiltrating inflammatory cells, or with LPS, mimicking the influx of bacterial components into the circulation. Given that propionate has been reported to suppress vascular endothelial cell activation (28), we performed these stimulations in the presence of propionate for 24 hours. Subsequently, cells were harvested, and *VCAM1* expression was evaluated using qPCR. Consistent with previous reports, we demonstrated that stimulation with IL-1β, TNF-α, or LPS increased *VCAM1* expression in HUVEC, and that this activation was suppressed by propionate (**Supplementary Figure 8A**).

To elucidate the mechanism underlying the propionate-mediated suppression of *VCAM1* expression, we focused on histone deacetylase (HDAC) inhibition, a known mechanism by which short-chain fatty acids exert anti-inflammatory effects (28). Western blot analysis showed that treatment with either propionate or trichostatin A (TSA, an HDAC inhibitor) moderately enhanced histone H3 acetylation in HUVEC, whereas stimulation with IL-1β or TNF-α alone had no such effect (**Figure 6B**). Furthermore, we confirmed that HDAC inhibition by TSA effectively suppressed the cytokine-induced upregulation of *VCAM1* expression (**Supplementary Figure 8B**), consistent with previous findings. Taken together, these results suggest that propionate suppresses inflammatory responses in vascular endothelial cells, potentially through mechanisms involving the inhibition of HDAC activity.

Next, we investigated the effect of propionate on the dedifferentiation of VSMC. In diseases closely related to vascular inflammation, vascular wall thickening is driven by excessive VSMC dedifferentiation (26). Because similar vascular wall thickening was observed in LMV pathology presenting with vasculitis (**Figure 3A**), we hypothesized that propionate suppresses VSMC dedifferentiation. To address this, human aortic smooth muscle cells (HAoSMCs) were cultured in a low-serum medium (1% FBS), which has been reported to induce the transition from a dedifferentiated to a differentiated phenotype (29). We confirmed that HAoSMCs, which generally maintain a dedifferentiated phenotype under standard culture conditions, still exhibited a dedifferentiated phenotype characterized by low α-smooth muscle actin (α-SMA) expression at 48 hours of low-serum culture, before fully differentiating by day 7. Although, vimentin is often evaluated as a dedifferentiation marker, its expression remained constitutively high and unaltered at both 48 hours and day 7 (**Supplementary Figure 9**). Thus, we utilized this 48-hour time point as a dedifferentiation model to evaluate the suppressive effects of propionate based on α-SMA dynamics. Short-chain fatty acids, such as butyrate, have been reported to inhibit VSMC dedifferentiation via HDAC inhibition (30). To verify this, we treated HAoSMCs with propionate or butyrate to determine whether propionate similarly suppresses dedifferentiation through this mechanism, and evaluated differentiation markers and histone acetylation 48 hours later. We demonstrated that both propionate and butyrate significantly upregulated α-SMA expression, indicating the suppression of the dedifferentiated phenotype (**Figure 6C**). Furthermore, western blot analysis showed a trend toward increased histone H3 acetylation following the addition of propionate (**Figure 6C**). These results suggest that propionate suppresses VSMC dedifferentiation, potentially through mechanisms involving HDAC inhibition. Taken together, these data suggest that propionate may protect against vascular wall thickening by preventing VSMC dedifferentiation at the local cellular level.

### 3.8 Propionate administration suppresses LMV pathology

**Figure 7:**
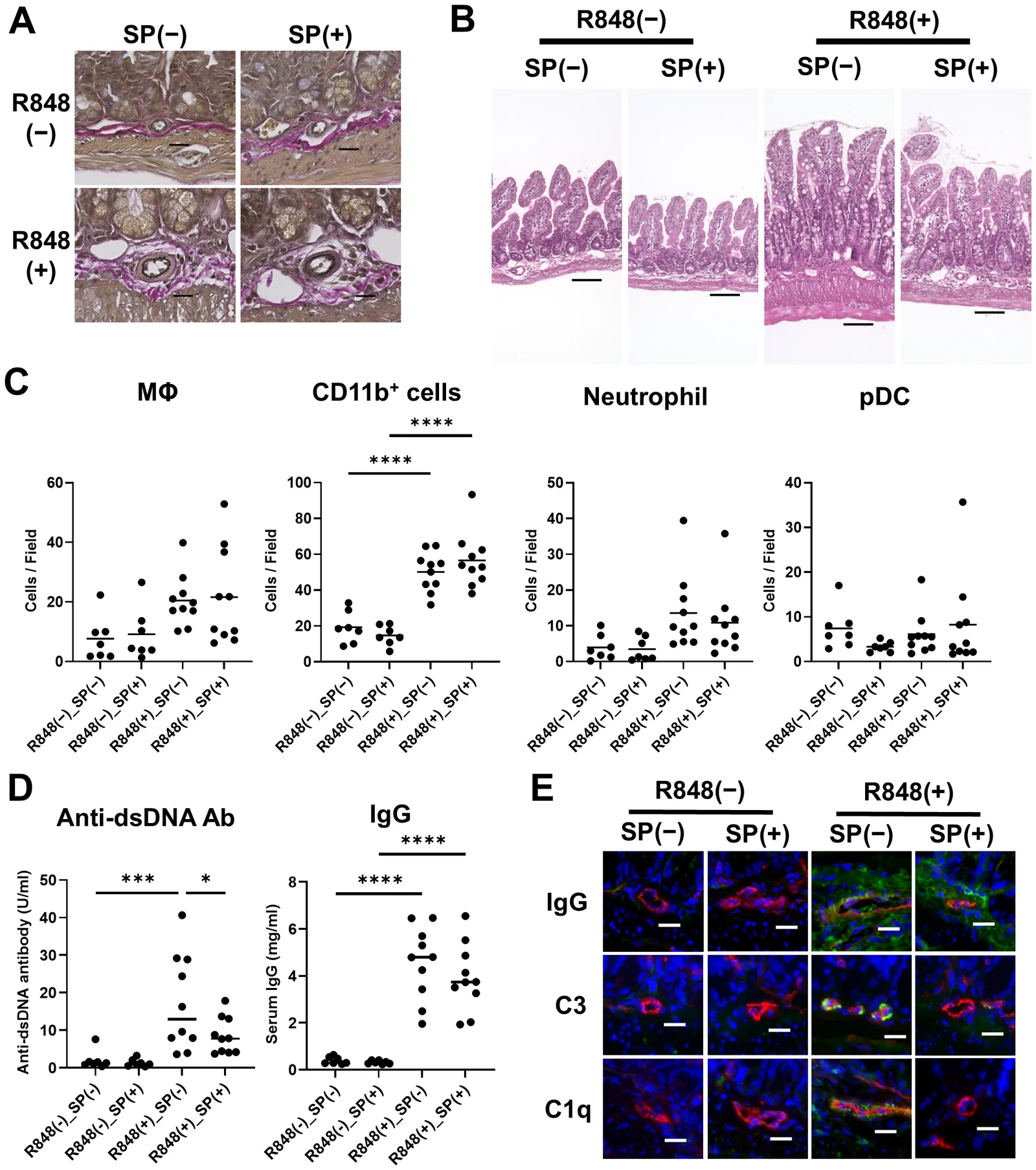
Propionate influences vascular inflammation in LMV pathology. Eight-week-old female BWF1 mice were treated topically with R848 on one ear three times weekly for 3 weeks. Sodium propionate (SP) administration was initiated simultaneously with R848 treatment and continued throughout the treatment period. (R848(−)_SP(−): *n* = 7, R848(−)_SP(+): *n* = 7, R848(+)_SP(−): *n* = 10, R848(+)_SP(+): *n* = 10). **(A)** Representative images of EVG-stained ileum sections from BWF1 mice. **(B)** Representative images of HE-stained ileum sections from BWF1 mice. **(C)** Inflammatory cell infiltration into lamina propria and submucosa of the ileum was analyzed using following markers: F4/80 (MΦ); CD11b (granulocytes, monocytes, macrophages, part of B cells, etc.); myeloperoxidase and Ly6G (neutrophils); and PDCA-1 and CD11c (pDC). The numbers of MΦ, CD11b^+^ cells, neutrophils, and pDC were counted up to three high-power fields (×200 magnification) per section. **(D)** Serum was collected and analyzed by ELISA for anti-dsDNA antibody and total IgG levels. **(E)** Representative images of ileum sections from BWF1 mice stained for IgG, C3, or C1q (green) and CD31 (red). Statistical analysis was performed using a one-way ANOVA followed by Sidak’s multiple-comparison test **(C, D)**. Each dot represents an individual mouse. Asterisks indicate statistically significant differences (*p < 0.05, ***p < 0.001, ****p < 0.0001).

Based on our *in vitro* data (**Figure 6**), we investigate the effect of propionate on LMV pathology by administering propionate in the drinking water of LMV-induced mice concurrently with disease induction and examined the pathology 3 weeks later. First, we assessed vasculitis using EVG staining of ileal blood vessels. Propionate administration tended to suppress vascular endothelial cell swelling (**Figure 7A**). However, HE staining of the ileum showed no change in perivascular cell infiltration following propionate administration (**Figure 7B**). In addition, we observed no changes in the infiltration of MΦ, CD11b^+^ cells, neutrophils, and pDCs, which frequently infiltrated in the submucosal and lamina propria after LMV induction (**Figure 7C**).

Regarding its anti-inflammatory effects, propionate has been reported to inhibit B cell differentiation and autoantibody production, thereby improving SLE pathology (31). Therefore, we investigated whether propionate administration suppresses SLE pathology in LMV-induced mice by examining its effects on serum IgG and anti-dsDNA antibody levels, which increase following LMV induction. Propionate administration significantly decreased anti-dsDNA antibody levels, and serum IgG levels also tended to decrease (**Figure 7D**). In addition, propionate treatment markedly suppressed the deposition of immune complexes in vascular endothelial cells observed upon LMV induction (**Figure 7E**). Furthermore, 6-week propionate administration suppressed vasculitis (**Supplementary Figure 10A**), which was likely due to the sustained suppression of immune complex deposition in blood vessels (**Supplementary Figure 10B**), indicating that this continuous effect is essential for propionate-mediated vasculitis inhibition. These results demonstrated that propionate plays a crucial role in suppressing vasculitis and SLE pathology. These findings suggest that increased propionate in the intestinal tract may maintain homeostasis and prevent the exacerbation of LMV pathology.

## 4 Discussion

LMV lacks characteristic clinical symptoms, and there are no established diagnostic indicators specifically for this disease. To address this, we established a novel induced LMV mouse model and investigated the mechanisms underlying the induction and exacerbation of LMV pathology.

Human LMV is characterized by thickening of the intestinal wall –whereas healthy individuals typically exhibit a thickness of 3 mm or less, LMV patients present with 8 mm or more– and vasculitis in the small blood vessels beneath the serosa (2, 4). In this study, we found that BWF1 mice treated with R848 exhibited more than a two-fold increase in intestinal wall thickness compared to the untreated group (**Figure 1A**). In addition, vasculitis was observed on the serosal side of the ileum (**Figure 3A**). Histological analysis demonstrated: 1) swelling due to the activation of vascular endothelial cells (**Figures 3A**, **5A**), and 2) thickening of the vascular wall due to the dedifferentiation of VSMC (**Figures 3A**, **5C**). Furthermore, we observed depositions of immune complexes on the vessel walls, specifically C1q deposition, which plays a crucial role as a hallmark of organ damage in SLE (**Figure 3B**). Antiphospholipid syndrome and thrombotic microangiopathy are known complications of SLE that present with findings similar to the vascular disorders observed in this study. Antiphospholipid syndrome is characterized by the production of anti-phospholipid antibodies (e.g., anti-cardiolipin antibodies) and vascular involvement with thrombocytopenia and thrombosis in the absence of vascular wall inflammation (32). In addition, C1q deposition is not observed in the vascular wall. In contrast, thrombotic microangiopathy is characterized by thrombocytopenia and renal dysfunction, accompanied by mild inflammation of the vascular wall due to thrombosis (33, 34). However, similar to antiphospholipid syndrome, C1q deposition is not observed in thrombotic microangiopathy. Therefore, these results suggest that the vasculitis observed in the ileum after R848 treatment is a vascular disorder distinct from antiphospholipid syndrome and thrombotic microangiopathy. Collectively, these findings demonstrate that this model recapitulates the pathogenesis of human LMV; thus, we established R848-treated BWF1 mouse as a novel induced LMV model. We further demonstrated that LMV pathology could be similarly induced by R848 treatment in MRL/*lpr* and BXSB mice, both of which possess a genetic predisposition to SLE (**Supplementary Figures 3A, B**). However, LMV pathology was not induced by R848 treatment in wild-type BALB/c mice (**Supplementary Figures 3C, D**). These results suggest that both a genetic predisposition to SLE and the activation of TLR7 by R848 may be necessary for the induction of LMV pathogenesis. Interestingly, in human LMV, the recurrence and mortality rates are higher when intestinal bleeding is observed in addition to intestinal wall thickening (5). Our model also exhibited bloody stools and intestinal bleeding (**Table 1**; **Figure 1C**; **Supplementary Figures 1A, C**), suggesting that this model mimics a clinical condition with a high recurrence rate and a poor prognosis.

In the LMV-induced mice, we observed decreased expression of tight junction-related genes (**Figure 1B**; **Supplementary Figure 7A**), which R848-treated mice has been reported to cause increased permeability of the intestinal epithelium (35). In addition, the expression of *Lypd8*, which suppresses the motility of *Escherichia coli* and flagellated bacteria, was also downregulated (**Supplementary Figure 7B**). These findings suggest that an environment conducive to bacterial translocation into the systemic circulation may be established. *In vitro* experiments showed that HUVEC were activated by stimulation with LPS, a component of invading bacteria (**Supplementary Figure 8A**). To verify the *in vivo* relevance of this finding, we treated the LMV-induced mice with a broad-spectrum antibiotic cocktail and found that the activation of vascular endothelial cells was suppressed (**Figure 5A**). In addition, we observed that the marked infiltration of inflammatory cells into the lesional area of the ileum (**Figure 4F**) and the dedifferentiation of VSMC were also reduced (**Figure 5C**). Based on these observations, we hypothesize the following sequential mechanism: 1) translocated bacterial components initially activate vascular endothelial cells; 2) this activation leads to increased vascular permeability and increased expression of adhesion molecules, thereby promoting the infiltration of inflammatory cells; and 3) these infiltrating cells secrete inflammatory cytokines, such as IL-1β and TNF-α. Importantly, systemic exposure to translocated bacterial components such as LPS, facilitated by intestinal barrier dysfunction, may further promote B cell activation and autoantibody production, thereby linking the initial barrier impairment to the subsequent autoimmune response (35). Thus, the activation of vascular endothelial cells and the dedifferentiation of VSMC (36) are likely driven by the combined actions of bacterial components and inflammatory cytokines. These findings suggest that the influx of these bacterial components may play a crucial role in inducing the initial pathogenesis of LMV. However, 6 weeks after R848 application, although vasculitis tended to be suppressed in the antibiotic-treated group (**Supplementary Figure 6E**), the deposition of immune complexes in blood vessels was observed (**Supplementary Figure 6F**). Thus, it is possible that vasculitis may subsequently progress due to these depositions. These results suggest that the direct involvement of bacteria in the process of LMV exacerbation and progression is limited; rather, the deposition of immune complexes may be the main factor driving disease progression.

Although their direct involvement in disease progression is limited, the intestinal microbiota play a crucial role in inducing the initial pathogenesis of LMV. To elucidate the specific functions of the intestinal microbiota in this pathological process, we next focused on the effects of altered bacterial metabolites associated with changes in the intestine microbiota. Analysis of fecal metabolites from LMV-induced mice demonstrated a significant increase in propionic acid (**Figure 6A**), suggesting enhanced propionate production in the intestinal tract. Therefore, we investigated the effects of increased propionate on blood vessels and LMV pathology. In *in vitro* experiments, propionate promoted histone H3 acetylation in HUVEC and HAoSMC (**Figures 6B, C**), suggesting the potential involvement of HDACs inhibition. This inhibition thereby suppressed HUVEC activation (**Supplementary Figure 8A**) and increased α-SMA expression in HAoSMC, inducing their differentiation (**Figure 6C**). These results are consistent with previous reports demonstrating the effects of short-chain fatty acids on vascular constituent cells (28, 30). Thus, these findings suggest that increased propionate in the intestines may suppress vasculitis by inhibiting the activation of vascular endothelial cells and inducing the differentiation of VSMC. To further investigate the role of increased propionate in LMV pathology, we administered propionate to the LMV-induced mice. We found that the deposition of immune complexes in blood vessels was significantly suppressed (**Figure 7E**; **Supplementary Figure 10B**), and the elevation of serum anti-dsDNA antibody levels was also inhibited (**Figure 7D**). This inhibition of autoantibody production is consistent with previous reports demonstrating that short-chain fatty acids, including propionate, suppress SLE pathogenesis by inhibiting B cell differentiation and autoantibody production (31, 37). In addition, because complement activation and leukocyte migration induce vascular injury, our findings suggest that the propionate-mediated suppression of immune complex deposition may have contributed to the attenuation of LMV progression (**Figure 7E**; **Supplementary Figure 10B**). While endogenous propionate was elevated in LMV-induced mice (**Figure 6A**), this physiological increase may only provide a modest mitigation of the pathology. Our results suggest that exogenous supplementation could bolster this limited compensatory response, helping to further attenuate disease progression. Thus, we believe that the R848-induced increase intestinal propionate might reflect a mild physiological response to suppress LMV pathogenesis, rather than a factor exacerbating the disease.

This study has several limitations. Although the LMV-induced mouse model recapitulated several aspects of human LMV pathology, some underlying mechanisms remain unclear. In the future studies, we will utilize this model to investigate these unidentified pathological conditions. Furthermore, although BWF1 mice, which spontaneously develop SLE, may also be susceptible to LMV, these mice develop SLE pathology in an age-dependent manner and exhibit highly diverse phenotypes, including but not limited to nephritis (18). Thus, verifying the spontaneous onset of LMV in this model may be time-consuming, and their phenotypic diversity could complicate the analysis. In addition, although our results suggest that activation of TLR7/8 signaling by R848, combined with a genetic predisposition to SLE, contributes to LMV pathogenesis, the precise individual roles of TLR7 and TLR8 remain to be fully clarified. While R848-driven immune responses in mice are generally attributed to TLR7 due to the relative hyporesponsiveness of murine TLR8 (38), R848 has been shown to physically bind to murine TLR8 and trigger signaling in non-immune cells (39). Thus, although TLR7 is likely the primary mediator of LMV, a potential contribution of TLR8 cannot be completely excluded. To address this, it is necessary to investigate the impact of blocking TLR7 and/or TLR8 signaling on LMV pathogenesis using knockout mice or neutralizing antibodies. Finally, to elucidate the mechanisms underlying LMV pathogenesis, this study focused on the intestinal microbiota and its metabolite, propionate. However, the intestinal environment is complicated and multifactorial; thus, it is necessary to investigate the involvement of other luminal factors in LMV pathogenesis, such as tryptophan metabolism, which has been reported to play a crucial role in SLE pathogenesis (40).

In conclusion, we demonstrated that continuous R848 application to mice with a genetic predisposition to SLE established a novel induced LMV model that recapitulates human LMV pathology. Our results suggest that the initial pathogenesis of LMV may be triggered by systemic immune activation, which is further promoted by the involvement of intestinal microbiota due to increased permeability of the intestinal epithelium. Furthermore, our findings suggest that the subsequent exacerbation of LMV may be driven by the deposition of immune complexes in blood vessels, a process potentially accelerated by increased autoantibody production following this immune activation. In addition, intestinal propionate levels increased following LMV induction; this increase likely reflects a modest physiological response to mitigate LMV pathogenesis and maintain homeostasis. Thus, utilizing this novel LMV mouse model could contribute to elucidating unidentified pathological mechanisms and discovering specific diagnostic markers and therapeutic targets for LMV.

## Data Availability

The datasets presented in this study can be found in online repositories. The names of the repository/repositories and accession number(s) can be found below: https://www.ebi.ac.uk/ena, PRJEB112358.

## In memoriam

We also wish to honor the memory of our late co-author and former principal investigator, Dr. Iwao Sekigawa, for his invaluable contributions to this study.
