# Supplementary material for: Establishment of a novel lupus mesenteric vasculitis mouse model and exploration of its exacerbation mechanisms
Source: Front Immunol. 2026 Sep 9;17:1886325. doi: 10.3389/fimmu.2026.1886325 (PMC13597410; doi:10.3389/fimmu.2026.1886325)
Supplement: Supplementary file 1 [file SupplementaryFile1.docx]

Supplementary Material

Table S1. Antibodies utilized in this study.

| Application | Fluorophore | Antigen | Clone | Company | RRID | Concentration |
| --- | --- | --- | --- | --- | --- | --- |
| FACS |  | CD16/32 | S17011E | BioLegend | AB_2783138 | 1:400 |
|  | PE | CD41 | MWReg30 | BioLegend | AB_2129745 | 1:40 |
|  | Biotin | Red blood cells | TER-119 | BioLegend | AB_313705 | 1:20 |
|  | Alexa Fluor 647 | Mouse IgG (H+L) | Polyclonal | Invitrogen | AB_2535805 | 1:500 |
|  | PerCP-Cy5.5 | Streptavidin |  | eBioscience |  | 1:100 |
| Immunofluorescence |  | C1q | JL-1 | Hycult biotech | AB_10128818 | 1:50 |
|  |  | C3 | 6A525 | Santa cruz | AB_1119820 | 1:500 |
|  |  | CD11b | M1/70 | BioLegend | AB_312785 | 1:2500 |
|  |  | CD11c | N418 | BioLegend | AB_313771 | 1:50 |
|  |  | CD31 (Goat) | Polyclonal | R&D | AB_2161028 | 1:100 |
|  |  | CD31 (Rat) | MEC 13.3 | BD Biosciences | AB_394816 | 1:100 |
|  |  | CD62P | Polyclonal | GeneTex | AB_10721871 | 1:200 |
|  |  | F4/80 | BM8 | Abcam | AB_443548 | 1:50 |
|  |  | Ly6G | 1A8 | BD Biosciences | AB_394206 | 1:250 |
|  |  | MPO | Polyclonal | Abcam | AB_307322 | 1:100 |
|  |  | PDCA-1 | 120G8 | DENDRITICS | AB_2827525 | 1:500 |
|  |  | Red blood cell | Polyclonal | Rockland | AB_220020 | 5 µg/ml |
|  |  | VCAM1 | 6C7.1 | GeneTex | *GTX54406 | 1:200 |
|  |  | Vimentin | D21H3 | Cell signaling | AB_10695459 | 1:400 |
|  |  | α-SMA | 1A4/ASM-1 | PROGEN | AB_2920672 | 1:250 |
|  | Alexa Fluor 647 | Goat IgG | Polyclonal | Invitrogen | AB_2535864 | 1:500 |
|  | Alexa Fluor 488 | Goat IgG | Polyclonal | Invitrogen | AB_2534102 | 1:500 |
|  | Alexa Fluor 488 | Hamster IgG | Polyclonal | Invitrogen | AB_2535759 | 1:500 |
|  | Alexa Fluor 488 | Mouse IgG (H+L) | Polyclonal | Invitrogen | AB_2534069 | 1:500 |
|  | Alexa Fluor 647 | Mouse IgG (H+L) | Polyclonal | Invitrogen | AB_162542 | 1:500 |
|  | Alexa Fluor 488 | Mouse IgM (µ chain) | Polyclonal | Invitrogen | AB_2535711 | 1:500 |
|  | Alexa Fluor 488 | Rabbit IgG | Polyclonal | Invitrogen | AB_2535792 | 1:500 |
|  | Alexa Fluor 594 | Rabbit IgG | Polyclonal | Invitrogen | AB_141637 | 1:500 |
|  | Alexa Fluor 488 | Rat IgG | Polyclonal | Invitrogen | AB_2535794 | 1:500 |
|  | Alexa Fluor 594 | Rat IgG | Polyclonal | Invitrogen | AB_2535795 | 1:500 |
|  |  | DAPI |  | DOJINDO |  | 1:500 |
| Western blotting |  | Vimentin | D21H3 | Cell signaling | AB_10695459 | 1:1000 |
|  |  | α-SMA | 1A4/ASM-1 | PROGEN | AB_2920672 | 1:500 |
|  |  | β-actin | AC-15 | Sigma-Aldrich | AB_476744 | 1:10000 |
|  |  | Actyle (Lys9)-Histone H3 | C5B11 | Cell signaling | AB_823528 | 1:1000 |
|  |  | Histone H3 | G3 | Abcam | AB_302613 | 1:1000 |
|  | Horseradish peroxidase | Mouse IgG | Polyclonal | Dako | AB_2636929 | 1:2000 |
|  | Horseradish peroxidase | Rabbit IgG | Polyclonal | Dako | AB_2617138 | 1:2000 |

*; Since RRID was not registered, the manufacturer's catalog number was provided to ensure traceability.

Table S2. Primers used for qPCR analysis.

Mouse primers

| Actb | F  R | 5’-  5’- | CATCCGTAAAGACCTCTATGCCAAC  ATGGAGCCACCGATCCACA | -3’  -3’ |
| --- | --- | --- | --- | --- |
| Cldn7 | F  R | 5’-  5’- | GGCCACTCGAGCCTTAATGGTG  CCTGCCCAGCCGATAAAGATGG | -3’  -3’ |
| F11r | F  R | 5’-  5’- | TGAAGACACTAATCCTCCTCTCTGC  GCTCCTCAGTTTTAGTCTCTTCATCTGTA | -3’  -3’ |
| Gapdh | F  R | 5’-  5’- | CAACTTTGTCAAGCTCATTTCCTG  CCTCTCTTGCTCAGTGTCCTT | -3’  -3’ |
| Icam1 | F  R | 5’-  5’- | AACTTTTCAGCTCCGGTCCTG  TCAGTGTGAATTGGACCTGCG | -3’  -3’ |
| Ifng | F  R | 5’-  5’- | CGGCACAGTCATTGAAAGCCTA  GTTGCTGATGGCCTGATTGTC | -3’  -3’ |
| Il1b | F  R | 5’-  5’- | TCCAGGATGAGGACATGAGCAC  GAACGTCACACACCAGCAGGTTA | -3’  -3’ |
| Il6 | F  R | 5’-  5’- | CCACTTCACAAGTCGGAGGCTTA  GCAAGTGCATCATCGTTGTTCATAC | -3’  -3’ |
| Il12a | F  R | 5’-  5’- | CATCGATGAGCTGATGCAGT  CAGATAGCCCATCACCCTGT | -3’  -3’ |
| Lypd8 | F  R | 5’-  5’- | GCCTTCACTGTCCATCTATTT  GTGACCATAGCAAGACATGCA | -3’  -3’ |
| Ocln | F  R | 5’-  5’- | AGACTACACGACAGGTGGGG  CTGCAGACCTGCATCAAAAT | -3’  -3’ |
| Tgfb1 | F  R | 5’-  5’- | GTGTGGAGCAACATGTGGAACTCTA  TTGGTTCAGCCACTGCCGTA | -3’  -3’ |
| Tjp1 | F  R | 5’-  5’- | GCAGACTTCTGGAGGTTTCG  CTTGCCAACTTTTCTCTGGC | -3’  -3’ |
| Tlr7 | F  R | 5’-  5’- | GGAGCTCTGTCCTTGAGTGG  CAAGGCATGTCCTAGGTGGT | -3’  -3’ |
| Tlr9 | F  R | 5’-  5’- | TCTCCCTTATGATGCCTTCGTG  TCCTCCAGACACAAGCGTAG | -3’  -3’ |
| Tnf | F  R | 5’-  5’- | GTTCTATGGCCCAGACCCTCAC  GGCACCACTAGTTGGTTGTCTTTG | -3’  -3’ |

Human primers

| ACTB | F  R | 5’-  5’- | TGGCACCCAGCACAATGAA  CTAAGTCATAGTCCGCCTAGAAGCA | -3’  -3’ |
| --- | --- | --- | --- | --- |
| VCAM1 | F  R | 5’-  5’- | TGCACAGTGACTTGTGGACATA  GCCACCACTCATCTCGATTT | -3’  -3’ |

Supplemental Figure S1. Resiquimod (R848) stimulation leads to intestinal wall thickening and bleeding in the small intestine of NZBWF1 (BWF1) mice.

Eight-week-old female BWF1 mice were treated with R848 three times weekly on one ear for 6 weeks. (A) Representative macroscopic images of the ileum. The left image shows R848(−) and the right image shows R848(+). Marked bleeding was observed in the area indicated by the arrow. (B, C) Representative images of Swiss roll preparations of the small intestine sections. In these preparations, the stomach side is on the inside, and the large intestine side is on the outside. Sections were stained (B) with hematoxylin-eosin (HE) and (C) for red blood cell (red). The white frame in (C) indicates the location of bleeding. Nuclei were stained with DAPI (blue). Scale bar = 1000 µm.

Supplemental Figure S2. Anti-neutrophil cytoplasmic antibodies (ANCA) are induced in BWF1 mice following R848 treatment.

Eight-week-old female BWF1 mice were treated with R848 for 6 weeks, and sera were collected. Mouse neutrophils were stained with these sera to detect the presence of ANCA (green). Nuclei were stained with DAPI (blue). Scale bar = 20 µm.

Supplemental Figure S3. Induction of lupus mesenteric vasculitis (LMV) pathology in different mouse strains and with another Toll like receptor (TLR) 7 agonist.

(A, B) Eight-week-old female BWF1 mice, six-week-old male BXSB mice, and five-week-old female MRL/*lpr* mice were treated with R848 for 3 weeks. Representative photos of ileum sections, stained with (A) HE and (B) elastica-van Gieson (EVG). Scale bar = 100 µm (A); 20 µm (B). (C, D) Eight-week-old female BALB/c mice were treated with R848 for 6 weeks. Ileum sections from BALB/c mice, stained with (C) HE and (D) EVG. Scale bar = 100 µm (C); 20 µm (D). (E, F) Eight-week-old female BWF1 mice were treated with imiquimod (IMQ) cream for 6 weeks. Histological analysis of ileum sections using (E) HE and (F) EVG staining. Scale bar = 100 µm (E); 20 µm (F).

**Supplementary Figure S4. R848-treated BWF1 mice changes intestinal microbiota.**

Eight-week-old female BWF1 mice were treated with R848 three times weekly on one ear for 6 weeks. Pooled fecal samples were collected and subjected to 16S rRNA sequencing analysis. (A) Bacterial diversity, as indicated by Shannon diversity index and Chao1 index. (B) PCoA plot showing the separation of overall bacterial community structures.

Supplemental Figure S5. Platelet counts and platelet-associated IgG levels in BWF1 mice after 3 weeks of LMV induction with and without antibiotics.

Eight-week-old female BWF1 mice were treated with R848 three times weekly on one ear for 3 weeks. An antibiotic cocktail comprising vancomycin, neomycin, ampicillin, and metronidazole was administered for 3 weeks prior to and continuously during the R848 treatment. (A) Platelet counts in whole blood from BWF1 mice were determined (R848(−)_ABS(−): *n*=33, R848(−)_ABS(+): *n*=17, R848(+)_ABS(−): *n*=38, R848(+)_ABS(+): *n*=25). (B) Platelet-associated IgG (PAIgG) levels were analyzed by flow cytometry using a fluorescently labeled anti-mouse IgG antibody, and the results are expressed as mean fluorescence intensity (MFI). See the “Materials and Methods” section for further details (R848(−)_ABS(−): *n*=7, R848(−)_ABS(+): *n*=7, R848(+)_ABS(−): *n*=15, R848(+)_ABS(+): *n*=15). Statistical analysis was performed using one-way ANOVA followed by Sidak’s multiple-comparison test*.* Each dot represents an individual mouse. Asterisks indicate statistically significant differences (*: *p* < 0.05, **: *p* < 0.01, ****: *p* < 0.0001). PLT: platelet; ABS: antibiotics.

Supplemental Figure S6. Phenotype of BWF1 mice after 6 weeks of LMV induction with and without antibiotics.

Eight-week-old female BWF1 mice were treated with R848 three times weekly on one ear for 6 weeks. Antibiotics were administered 3 weeks before the start of R848 treatment and continuously during R848 treatment (R848(−)_ABS(−): *n*=10, R848(−)_ABS(+): *n*=10, R848(+)_ABS(−): *n*=10, R848(+)_ABS(+): *n*=10). (A) Spleen weights from BWF1 mice are shown. (B, C) Serum total IgG (B) and anti-double-stranded DNA (dsDNA) antibody (C) levels were measured. (D, E) Representative images of ileum sections stained with (D) HE and (E) EVG are shown. (F) Representative immunofluorescence images of ileum sections stained for IgG, C3, or C1q (green) and CD31 (red) are shown. Nuclei were counterstained with DAPI (blue). Scale bar = 100 µm (D) and 20 µm (E, F). Statistical analysis was performed using one-way ANOVA followed by Sidak’s multiple-comparison test (A-C)*.* Each dot represents an individual mouse. Asterisks indicate statistically significant differences (*: *p* < 0.05, **: *p* < 0.01, ***: *p* < 0.001, ****: *p* < 0.0001). ABS: antibiotics.

Supplemental Figure S7. Effects of antibiotic administration on intestinal barrier-related gene and the liver in LMV mice.

Eight-week-old female BWF1 mice were treated with R848 three times weekly on one ear for 3 weeks. Antibiotic administration was initiated 3 weeks prior to and continued throughout the R848 treatment period. (A, B) Ileal tissues were collected from BWF1 mice, total RNA was extracted, and the expression levels of (A) *Cldn7* and (B) *Lypd8* were determined by quantitative polymerase chain reaction (qPCR). Gene expression levels were normalized to *Gapdh* expression (R848(−)_ABS(−): *n*=7, R848(−)_ABS(+): *n*=7, R848(+)_ABS(−): *n*=14, R848(+)_ABS(+): *n*=15). (C) Liver weights of BWF1 mice (R848(−)_ABS(−): *n*=5, R848(−)_ABS(+): *n*=5, R848(+)_ABS(−): *n*=11, R848(+)_ABS(+): *n*=11). (D) Representative images of liver sections from BWF1 mice stained with HE and Elastica Masson (EM). Scale bar = 100 µm. Statistical analysis was performed using a one-way ANOVA followed by Sidak’s multiple comparison test (A-C)*.* Each dot represents an individual mouse. Asterisks indicate statistically significant differences (**: *p* < 0.01, ***: *p* < 0.001, ****: *p* < 0.0001). ABS: antibiotics.

Supplemental Figure S8. Effects and mechanisms of propionate on vascular endothelial cells *in vitro*.

(A) Human umbilical vein endothelial cells (HUVEC) were exposed to IL-1β, TNF-α, or lipopolysaccharide (LPS) with or without sodium propionate (SP) for 24 h. *VCAM1* expression levels were examined by qPCR and normalized to that of *ACTB* (*n*=5). (B) HUVEC were exposed to IL-1β or TNF-α with or without trichostatin A (TSA) for 6 h. *VCAM1* expression levels were examined by qPCR and normalized to that of *ACTB* (*n*=4). Statistical analysis was performed using a one-way ANOVA followed by Sidak’s multiple comparison test. Each dot represents an individual assay. Asterisks indicate statistically significant differences (*: *p* < 0.05, ***: *p* < 0.001, ****: *p* < 0.0001).

Supplemental Figure S9. Effects of culture environment on vascular smooth muscle cells.

Human aortic smooth muscle cells (HAoSMC) were cultured for 48 h and 7 days, and total protein was extracted. The left panel shows representative western blots for vimentin and alpha-smooth muscle actin (α-SMA). β-actin expression is shown as a loading control. The graphs show a densitometric analyses of the bands (*n*=4). Statistical analysis was performed using a two-tailed Student’s *t*-test*.* Each dot represents an individual assay. Asterisks indicate statistically significant differences (***: *p* < 0.001).

Supplemental Figure S10. Effects of propionate for 6 weeks on blood vessels *in vivo*.

Eight-week-old female BWF1 mice were treated with R848 three times weekly on one ear for 6 weeks. Sodium propionate (SP) administration was initiated simultaneously with the R848 treatment and continued throughout the experimental period (R848(−)_SP(−): *n*=3; R848(−)_SP(+): *n*=3; R848(+)_SP(−): *n*=3; R848(+)_SP(+): *n*=3). (A) Representative images of ileum sections from BWF1 mice stained with EVG. Scale bar = 20 µm. (B) Representative images of ileum sections from BWF1 mice stained for IgG, C3, or C1q (green), and CD31 (red). Nuclei were stained with DAPI (blue). Scale bar = 20 µm.
